# Identification of a novel metastasis inducing lncRNA which suppresses the KAI1/CD82 metastasis suppressor gene and is upregulated in triple-negative breast cancer

**DOI:** 10.18632/oncotarget.18733

**Published:** 2017-06-28

**Authors:** Ronni Aram, Iris Dotan, Agnes Hotz-Wagenblatt, Dan Canaani

**Affiliations:** ^1^ Department of Biochemistry and Molecular Biology, George Wise Faculty of Life Sciences, Tel Aviv University, Ramat Aviv 69978, Israel; ^2^ Bioinformatics Group, Core Facility Genomics & Proteomics, German Cancer Research Center (DKFZ), D-69120 Heidelberg, Germany

**Keywords:** lncRNA, metastasis suppressor genes, triple-negative breast cancer, epigenetic gene regulation, lncRNA and metastasis

## Abstract

Inactivation of tumor/metastasis suppressor genes via epigenetic silencing is a frequent event in human cancers. KAI1/CD82 is a metastasis suppressor gene whose normal protecting activity is deficient in twelve different solid malignancies. Here we have identified and characterized a primarily nuclear non-polyadenylated, antisense (as)-lncRNA, initiating upstream of the KAI1 human metastasis suppressor gene transcription start site; and elongating in the opposite direction to KAI1 mRNA. We show that the KAI1 promoter is bi-directional giving rise to KAI1 mRNA and its as-lncRNA. Moreover, expression of this lncRNA transcript emerges to be inversely related to the KAI1 mRNA expression, and in direct relationship to the invasiveness level of human breast cancer derived cell lines. Importantly, knockdown of the KAI1 as-lncRNA in the triple-negative breast cancer cell line MDA-MB-231 have led to increased KAI1 mRNA and protein expression, manifested in stronger adhesion to fibronectin, retardation of cell migration and reduced cell invasion *in vitro*. Accordingly we have named this lncRNA, SKAI1BC, standing for “Suppressor of KAI1 in Breast Cancer”. These results uncover a potential way to harness tumor metastasis via targeting SKAI1BC in human breast cancer, and perhaps also in other KAI1-deficient human malignancies.

## INTROUCTION

Curiously, only a small percentage of cancer causations are attributed to variations of protein coding sequences, while more frequent being changes in gene expression levels [1]. Recently, growing evidence indicate that lncRNAs are involved in tumorigenesis by showing aberrant expression in cancer cells in comparison to healthy tissue cells [1, 2, 3]. Two early on examples has to do with the tumor suppressors p15 and p21 and their promoters’ spanning antisense lncRNAs (p15-as; p21-as). Ectopic expression of siRNAs against the natural promoter spanning antisense lncRNAs of p15 and p21, have re-activated these transcriptionally silenced tumor suppressor genes, which indicates oncogenic features of these antisense lncRNAs [4, 5]. These latter results point toward the potential of promoter-directed shRNAs to activate gene expression of tumor/metastasis suppressor genes silenced by promoter spanning as-lncRNAs. Surprisingly, divergent transcription that results in a sense mRNA and an antisense lncRNA (as-lncRNA) has been observed in ∼50-80% of human protein coding gene promoters [6]. These bi-directional promoters are associated among others with neuronal functions, regulation of tumor suppressors and oncogenes [7].

The KAI1 metastasis suppressor gene (also known as CD82 or Tspan27), is located on human chromosome 11p11.2 and encodes for a 267 amino acid transmembrane protein that belongs to the tetraspanin family. According to the Ensembl gene browser, the KAI1 transcript can be found in 14 splice variants of which only the main variant leads to translation of the functional protein. Numerous *in vitro* studies have shown that KAI1 over-expression inhibits cell motility and invasion [8]. Tumor cells must detach from the cell mass in order to invade adjacent tissue. The ability to invade is associated with the transition of cell-cell and cell-ECM adhesion molecules. It has been suggested that KAI1 has the ability to reorganize the assembly of membrane proteins and molecular concentration of integrins, which modulate the adhesive strength of the cell and promotes cell aggregation [8]. As a metastasis suppressor, KAI1 has not only the task to suppress cell motility but also to prevent invasion of tumor cells by inactivating proteases that degrade the extracellular matrix. KAI1 causes a redistribution of urokinase plasminogen activator surface receptor (uPAR) and α5β1 integrins. This redistribution results in macromolecular assemblies that prevent uPAR from binding its ligand urokinase-type plasminogen activator (uPA) and subsequently in a reduced ECM proteolysis. KAI1 has been identified as a metastasis suppressor in human prostate, melanoma, sarcoma, pancreatic and breast cancer cell lines. A direct correlation of a good prognosis and KAI1 expression has been observed in the following solid tumors: melanoma, non-small cell lung, breast cancer [reviewed in ref. 8]. Noteworthy, in at least three solid tumors (gastric, cervical, and ovarian cancers) KAI1 affects not only tumor metastasis but also tumor proliferation. In the case of breast tumors, KAI1 expression is clearly significantly reduced during cancer progression [9]. At the transcriptional level KAI1 is upregulated by several transcription factors such as AP2, p53, JunB, and ΔNp63α. Post transcriptionally, in HCC cells KAI1 is negatively regulated via miR-197 interaction with its 3' UTR sequence (8). In several human melanoma cell lines, there is loss of heterozygosity (LOH) of the 11p11.2 region which contains the KAI1 gene as well. However, LOH of KAI1 in human cancers is a rare event, and similarly no point mutations have been found in the KAI1 gene in human malignancies (8). Thus, it is being assumed that KAI1 expression in human tumors is being epigenetically silenced by additional mechanisms.

In this work, through careful RT-PCR analysis of candidate bidirectional promoters among human genes encoding tumor suppressors and metastasis suppressors known to affect breast cancer, we identified a novel lncRNA. Characterization of this nuclear antisense lncRNA, spanning the promoter/enhancer region of the KAI1/CD82 metastasis suppressor gene, has shown that it is a suppressor of the KAI1 gene. Expression of this lncRNA is inversely related to the KAI1 expression, and in direct relationship to the invasiveness level of human breast cancer derived cell lines. As KAI1 is a metastasis suppressor gene in at least 12 solid human tumors, it would be extremely desirable to target this suppressing lncRNA, in the hope to retard or halt cell metastasis all together.

## RESULTS AND DISCUSSION

### Screening for promoter-spanning lncRNAs of metastasis/tumor suppressor genes in triple-negative breast cancer cell lines

In order to try identifying new breast cancer affecting lncRNA(s), we followed Morris [4] and Yu [5] example, by screening for promoter-spanning lncRNAs in metastasis- and/or tumor-suppressor genes, in which reduced transcript/s is the basis for the loss of their gene expression. To this end, the extent of mRNA expression in ten metastasis-/tumor-suppressor genes was analyzed by semi-quantitative RT-PCR in three different triple-negative breast cancer (TNBC) cell lines, namely MDA-MB 231, Hs578T and SUM149PT. Upon analysis of these three cell lines, expression of the genes FKBP4, KIF1A and OGDHL seemed to be high in all three. The high transcript levels are likely to indicate a lack of negative transcription regulation in TNBC cell lines and hence these genes were excluded from screening for promoter spanning lncRNA (which might have impeded their gene expression). Contrary, the expression of Cst6, MAL, VGF, RARbeta, Maspin, SYK and KAI1 genes varied, depending on the particular cell line examined. These differences in gene expression might indicate a cell line specific transcriptional regulation.

**Figure 1:**
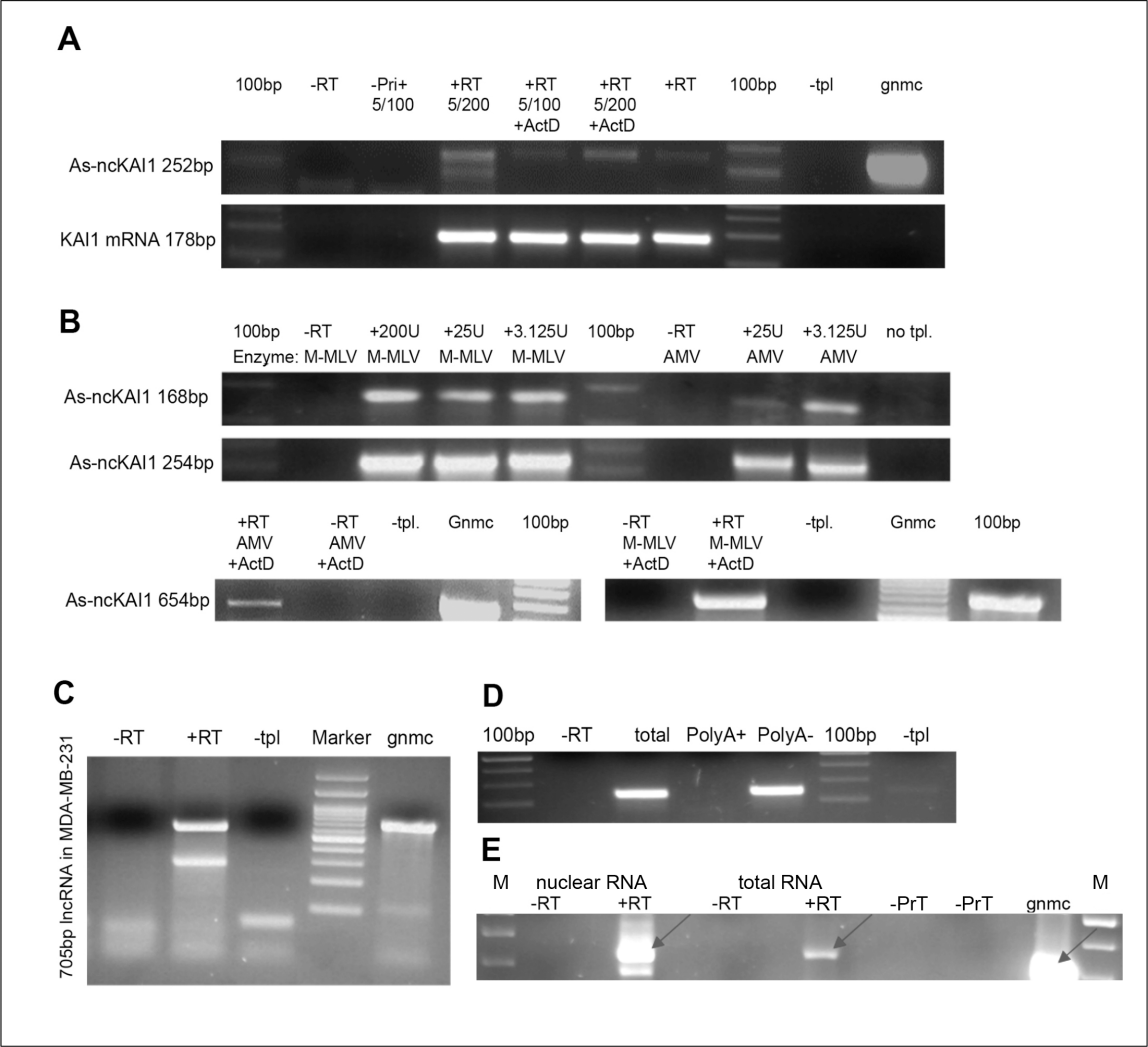
KAI1 as-lncRNA; analysis via RT-PCR **(A)** Products obtained in the presence of actinomycin D in the RTase reaction. Total MDA-MB-231 RNA has been reverse transcribed with M-MLV RTase with/without 50ng/μl Actinomycin D, followed by PCR amplification after dilution of Actinomycin D to 2.5 ng/μl (5/100) or 1.25ng/μl (5/200). **(B)** Dependence on particular RTase? Total MDA-MB-231 RNA was reverse transcribed with either M-MLV RTase or AMV RTase, and a transcript specific RT-primer (upper) or hexamers (lower), followed by PCR with Hot start Taq-Polymerase. **(C)** 5’ end mapping. MDA-MB-231 RNA treated by DNase and periodate was reverse transcribed with specific RT5 primer (+RT). PCR amplification led to 705 bp product. **(D)** 3’ end polyadenylation? Polyadenylated MDA-MB-231 RNA was pulled down with magnetic beads, while polyA minus-RNA remained in the flow through fraction. Both fractions and total RNA were reverse transcribed with KAI1 as-lncRNA specific primer. PCR amplifies a 168 bp product. **(E)** nuclear localization MDA-MB-231 RNA (2ug nuclear or total RNA fraction) was reverse transcribed with KAI1 as-lncRNA specific primer in presence (+RT) or absence (−RT) of RTase. 317 bp amplified PCR product indicated with an arrow. **Abbreviations:** –RT= no RT enzyme. –Primer/ -Pri (+)= no primer in RT reaction. –PrN = no primer in nuclear RNA fraction; -PrT=no primer in total RNA fraction. +RT= with RT enzyme. –tpl= no template. +ActD= 50ng/ μl Actinomycin D in RT reaction. 100 bp= DNA ladder. M= DNA marker; gnmc= genomic DNA.

To identify lncRNAs, total cellular RNA/nuclear RNA (isolated as outlined in Materials and Methods), was subject to site/primer-specific RT-PCR. To prevent PCR amplification of genomic DNA, the breast cancer cell line extracted RNA was exhaustively treated with DNase. Also, in order to prevent the endogenous RNAs from serving as primers in the RT reaction, the RNA samples were treated with sodium periodate which blocked free 3' RNA ends from serving as endogenous primers in the RT reaction, as we described in Tzadok et al. [10]. The reverse transcription was performed at elevated temperatures (50-60°C) to prevent false priming. Moreover, the RT reactions were done in the presence of Actinomycin D to prevent the synthesis of the second DNA strand, which may falsely indicate the presence of antisense RNA [11]. However, since Actinomycin D is also a DNA- Polymerase inhibitor, its concentration was diluted to an insignificant amount prior to the PCR reaction step. Noteworthy, out of these seven tested genes only one, KAI1, had an antisense lncRNA spanning its promoter in MDA-MB-231 cells (Figure 1A).

In order to rule out the possibility that for the KAI1 as-lncRNA a reverse transcriptase template switching [“RT jump”, 12] has occurred, thereby generating a false positive transcript, total RNA was reverse transcribed with two different reverse transcriptase (RTase) enzymes: a recombinant M-MLV RTase with a reduced RNase H activity and an AMV RTase (with RNase H activity). The putative KAI1 as-lncRNA could be detected in RNA reverse transcribed with both RT-enzymes, (Figure 1B), increasing the level of certainty that the product is not based on a template switching event (DNA to RNA or RNA to RNA).

### Structural characterization and subcellular localization of the KAI1 as-lncRNA

As the KAI1 promoter spanning as-lncRNA proved itself as a *bona fide* long noncoding RNA, a further study concentrated on its physical and functional characterization. In order to determine the 3' end of this lncRNA transcript, RT-PCR “walks” on the KAI1 as-lncRNA transcript towards its 3' end were performed, as graphically presented in Supplementary Figure 1. The primer RT5 (CAACGGTGTGTTGTGAGAGG) anneals −1180bp upstream of the KAI1 mRNA transcription start site (TSS), specifically, the major TSS, and is the furthest RT primer 5' of the TSS with which an antisense transcript was detected (see Supplementary Figure 1). The amplified 705 bp product of the RT-PCR reaction with the primers pair RT5 and Promoter-3-R (ATTCCAGGGCGGGTGTAT) is shown in Figure 1C.

The 5' end of the KAI1 as-lncRNA was then determined by performing a 5'-RACE reaction (See M & M). Here, a specific oligomer (TCAGCGCTTGGCATAGAG) primed the reverse transcription of the KAI1 as-lncRNA. Afterwards, the generated cDNA was tailed with poly(dT) by a terminal transferase, which enabled the PCR primer with a poly-A leader sequence to anneal, thereby allowing the transcript to be amplified. Next, the products of a Nested-PCR have been cloned and sequenced. The sequencing revealed that the 5'-end of the transcript is located at position −386 of the KAI1 mRNA TSS. Interestingly, the detected 5' end, as well as the rest of the sequence are identical to the sequence of a 792 bp long RNA transcript (UCSC Accession wgEncodeEH000148), found through sequencing of the GM12878 whole cell PolyA minus RNA fraction (contig_343318). A product downstream of The 3'-end of the indicated 792bp transcript was not detected by RT-PCR, or by priming the transcript with oligo dT (Figure 1C), thus suggesting absence of a poly-A tail. Therefore, the RNA transcript UCSC Accession wgEncodeEH000148 and the KAI1 as-lncRNA transcript which was identified herein are the same. However, no other information beside the sequence was known/published about this RNA transcript.

It has been reported that only 17.8% of the human lncRNAs are polyadenylated. In order to test whether the antisense transcript of the KAI1 (spanning the promoter and enhancer regions identified herein) is polyadenylated or not, polyadenylated MDA-MB-231 RNA was pulled down with Oligo (dT)_25_ - biotinylated Dynabeads ^®^ (as outlined in M & M), and RT-PCR was performed on the PolyA plus as well as on the flow through (PolyA minus) fraction. The KAI1 as-lncRNA transcript could only be detected in the PolyA minus flow through fraction (Figure 1D). This implies that the transcript is not polyadenylated.

In order to estimate the subcellular distribution of the KAI1 as-lncRNA, a semi-quantitative analysis of the RT-PCR product has been performed. Accordingly, 2 μg of nuclear RNA and 2 μg of total RNA have been reverse transcribed and amplified by PCR. The signal in the total RNA sample was much weaker than in the nuclear sample (Figure 1E), suggesting an enriched KAI1 as-lncRNA fraction in the nucleus.

### Human KAI1 as-lncRNA: transcript sequence homology in mammalian species

An extensive search of homologous transcripts in other species turned out to be fruitful. In the transcriptome shotgun sequencing database, three mammalian transcripts from Equus asinus, Equus przewalskii and Bubalus bubalis have shown high homology to the KAI1 as-lncRNA transcript (Supplementary Figure 2). When superimposing these top 3 homologous transcripts with the KAI1 as-lncRNA sequence, a 78 bp long sequence, which is mutual to all four transcripts, can be identified (Supplementary Figure 2). Its complementary sense sequence has been reported to be a regulatory region (enhancer) of the KAI1 gene, and contains binding motifs for three transcription proteins, AP1, AP2 and p53 [13]. However, the triple-negative cell lines which we use are deficient in wt. p53 expression. The finding of this partial homology fits the observations that contrary to short ncRNAs, lncRNAs are not always highly conserved but can be enriched in conserved sequence motifs [14–16].

### The KAI1 bi-directional promoter

As outlined above, the 5' end of the KAI1 as-lncRNA has been identified by 5' RACE and is located in proximity to the canonical KAI1 mRNA TSS. Since the transcript also spans the enhancer region of the KAI1 gene, and might be involved in transcription regulation of the KAI1 mRNA, it was examined whether a coordinately expressed gene pair transcripts, composed of the KAI1 mRNA and KAI1 as-lncRNA are present. In other words, it was examined whether this TATA- less promoter initiates transcription in both directions.

**Figure 2:**
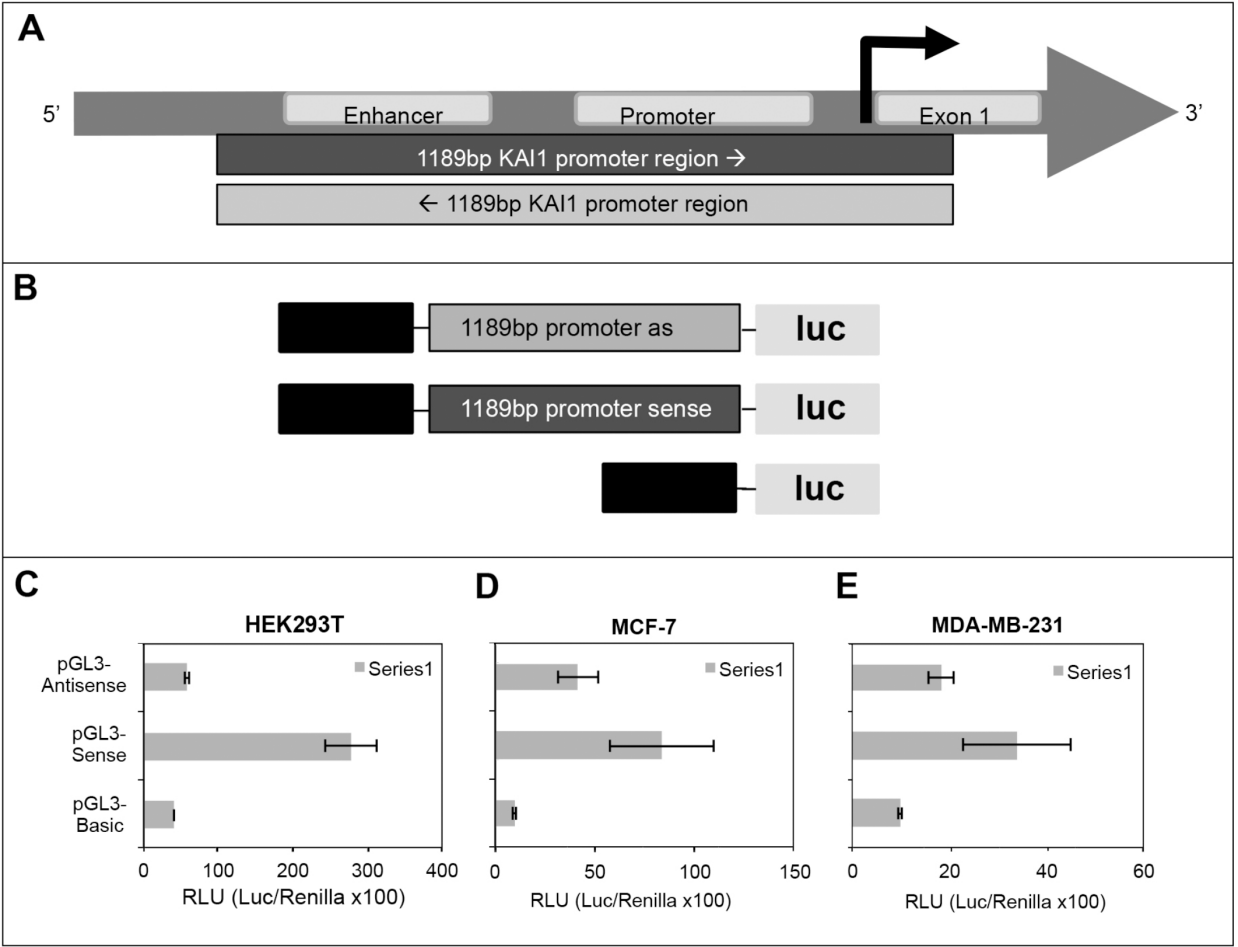
Testing KAI1 for having a bidirectional promoter via dual-luciferase assay **(A)** 1198 bp test region relative to the KAI1 gene map. **(B)** pGL3-firefly luciferase constructs. Upper: pGL3-firefly luc-basic-1198 bp KAI1 promoter in antisense orientation; middle: pGL3-firefly luc-basic-1198 bp KAI1 promoter in sense orientation; lower: pGL3-firefly luc-basic. **(C)** Dual-luciferase assay in HEK293T cells. **(D)** Dual-luciferase assay in MCF-7 cells. **(E)** Dual-luciferase assay in MDA-MB-231 cells. The dual-luciferase assays were performed as described in detail in M & M.

Toward this goal the pGL3-basic Vector (Promega) was digested with SmaI and blunt-end ligated with the PCR amplified (Q5 polymerase) 1198 bp KAI1 promoter-enhancer region (according to Marreiros et al., in ref. 13); having nucleic acid sequences bounded by the upstream sense sequence GTCAGCTGCACAGCTGAATG, and the downstream antisense sequence ACAGCCCGGGGCTCAGTCAC, respectively, as schematically shown in Figures 2A and 2B. The model cells HEK293T were co-transfected by pGL3-firefly luciferase-1198 bp promoter-sense or -antisense orientation, and pTK-Renilla luciferase DNA as an internal normalizing control (see M & M). Importantly, as shown in Figure 2C the promoter in the antisense orientation initiated firefly luciferase transcription, albeit at a ∼4.7 fold weaker level than the promoter in the sense orientation (i.e. orientation of the KAI1 mRNA). The dual-luciferase assay was also performed in MDA-MB-231 and MCF-7 cells, which showed as expected a poorer transfection rate, but still indicated a bidirectional activity of the KAI1 promoter (Figures 2D and 2E). In MDA-MB-231 and MCF-7 cells the promoter activity in the sense orientation is only about twice as high as in the antisense orientation. These results indicated that the KAI1 promoter is active in both orientations, manifesting a bidirectional regulation of the KAI1 mRNA and KAI1 as-lncRNA. This finding is not surprising in view of the fact that the KAI1 promoter is known to be GC-rich and to lack a TATA-box. The latter are very common among bidirectional promoters, since the TATA-box usually regulates the directionality of the transcription [17, 18].

### KAI1 as-lncRNA expression in breast cancer cell lines

**Figure 3:**
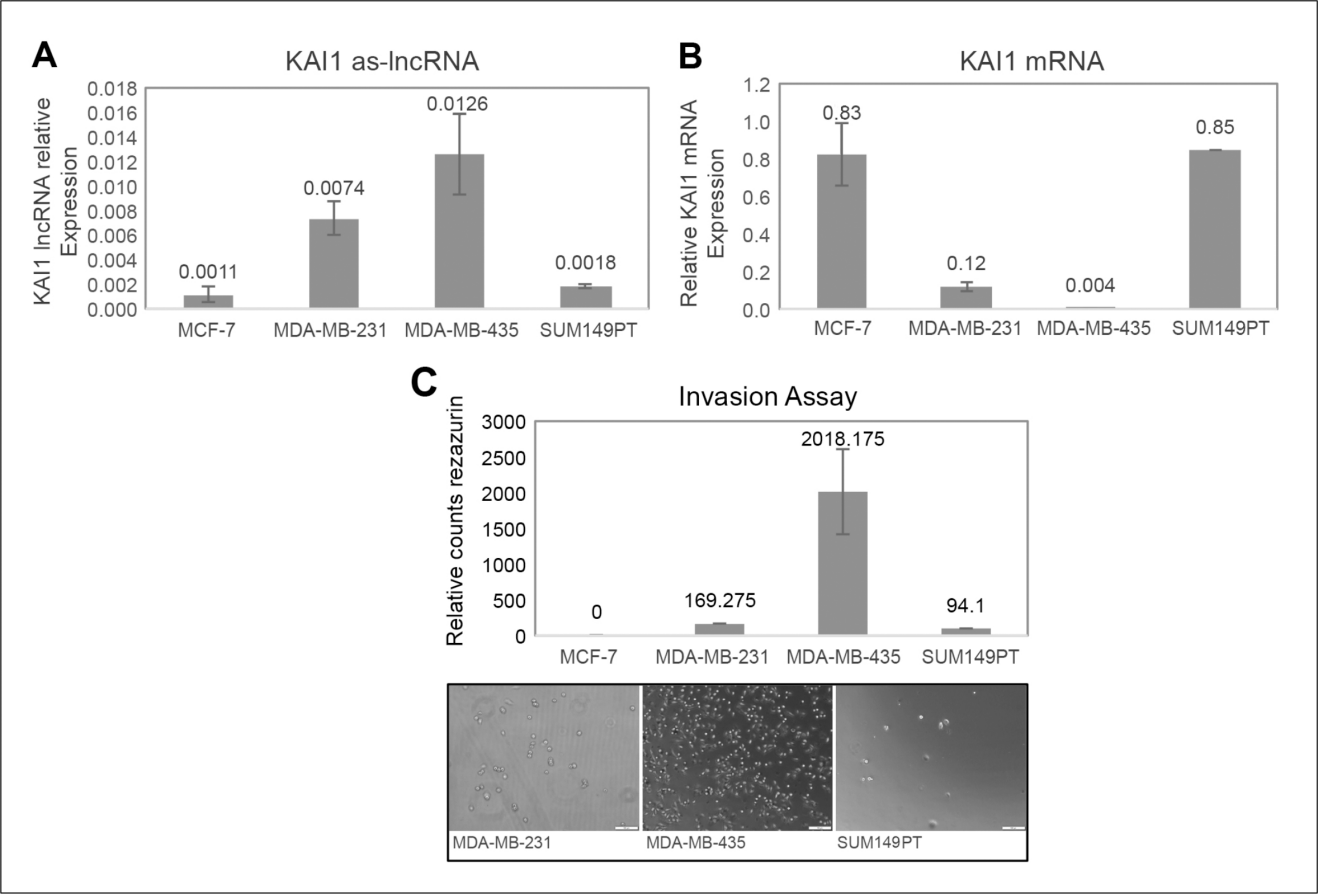
Expression in breast cancer cell lines of KAI1 as-lncRNA, KAI1 mRNA, and comparison of their *in vitro* invasion potency **(A)** MCF-7, MDA-MB-231, MDA-MB-435 and SUM149PT cell lines derived RNAs were subjected to DNase. Then 1μg RNA of each was reverse transcribed with RevertAid Premium followed by quantitative Real-Time PCR method. **(B)** 1μg RNA of each of the cell lines was reverse transcribed using oligodT_(15)_ priming. KAI1 mRNA levels relative to endogenous control HPRT-1 mRNA were evaluated by quantitative Real-Time PCR. **(C)** The four indicated cell lines were seeded in Matrigel coated transwell chambers. Cells invading from upper chamber to the chemo attractant (10% serum) in the lower chamber were detected using resazurin cell viability assay. Images of random fields were taken by light-field microscope camera as illustrative support.

The expression level of the KAI1 as-lncRNA was tested by qRT-PCR (see M & M) in two TNBC cell lines (MDA-MB-231 and SUM149PT), in the luminal breast cancer cell line MCF-7, and in the breast triple-negative/M14 melanoma derived MDA-MB-435 cell line. The relative KAI1 as-lncRNA expression vs. KAI1 mRNA expression was compared in order to evaluate a possible correlation to the putative bi-directional promoter expression. As shown in Figure 3A, while the KAI1 as-lncRNA expression in MCF-7 and SUM149PT is barely detectable via qRT-PCR, the transcript was more abundant in MDA-MB-231 and MDA-MB-435 cells. Next, the KAI1 mRNA expression level was determined in the above four cell lines, while HPRT-1 mRNA served as an endogenous control. As shown in Figure 3B, a relatively high KAI1 mRNA Expression level was detected in MCF-7 and SUM149PT cells, whereas in MDA-MB-231 cells the KAI1 mRNA expression was found to be very low, and in MDA-MB-435 the expression was barely detectable.

An inverse correlation between KAI1 expression and the metastatic potential of breast cancer cells was previously described, suggesting that KAI1 is a metastasis suppressor gene [9]. The capability of a cell to be metastatic can be measured based on its invasiveness. Therefore, the invasiveness of the four indicated cell lines was studied using a classical invasion assay, as outlined in M & M.

As demonstrated in Figure 3C, MDA-MB-435 appears to take the lead in the invasiveness, followed by MDA-MB-231 and SUM149PT. MCF-7 cells were not invasive enough to be observed and fell under the detection barrier. In these four cell lines, it is suggested that a high KAI1 as-lncRNA expression coordinates with a low KAI1 mRNA level, leading to high invasiveness.

### Knockdown of KAI1 as-lncRNA

Realizing the inverse relationship between the KAI1 as-lncRNA expression and the KAI1 mRNA level as demonstrated above, we tested whether manipulation of the KAI1 as-lncRNA level would affect the KAI1 mRNA expression. In order to test if the KAI1 as-lncRNA regulates the transcription or stability of its associated KAI1 mRNA, an shRNA encoding fragment directed against the KAI1 as-lncRNA (shRNA-1) was cloned into the lentiviral plasmid SHC203 (Sigma). Cell transduction with the SHC203-shRNA constructs was performed by virus infection (see M & M). A scrambled shRNA served as a “non-silencing” negative control, while an shRNA-less plasmid has been an empty-vector negative control. Noteworthy, it has been well documented that RNA interference can also occur in the nucleus [reviewed in ref. 19, and “Avivi S., Mor A., Dotan I., Kanter I., Tzadok S., Canaani D., Shav Tal Y.”, submitted for publication).

In order to ensure that the shRNA directed against the KAI1 as-lncRNA indeed leads to knockdown of the latter transcript, MDA-MB-231, MDA-MB-435 and MCF-7 cells were infected with the lentivirus encoding the shRNA-1 directed against the KAI1 as-lncRNA. Since the primers pair which amplifies the KAI1 as-lncRNA, could in the absence of an intron give rise to the same fragment size also from genomic DNA, an exhaustive DNA digestion of the RNA preparations had to precede the qRT-PCR assay. One more precaution was made to include also a “minus RT” control; lack of a product in the latter assured the total digestion-elimination of genomic cellular DNA (data not shown).

**Figure 4:**
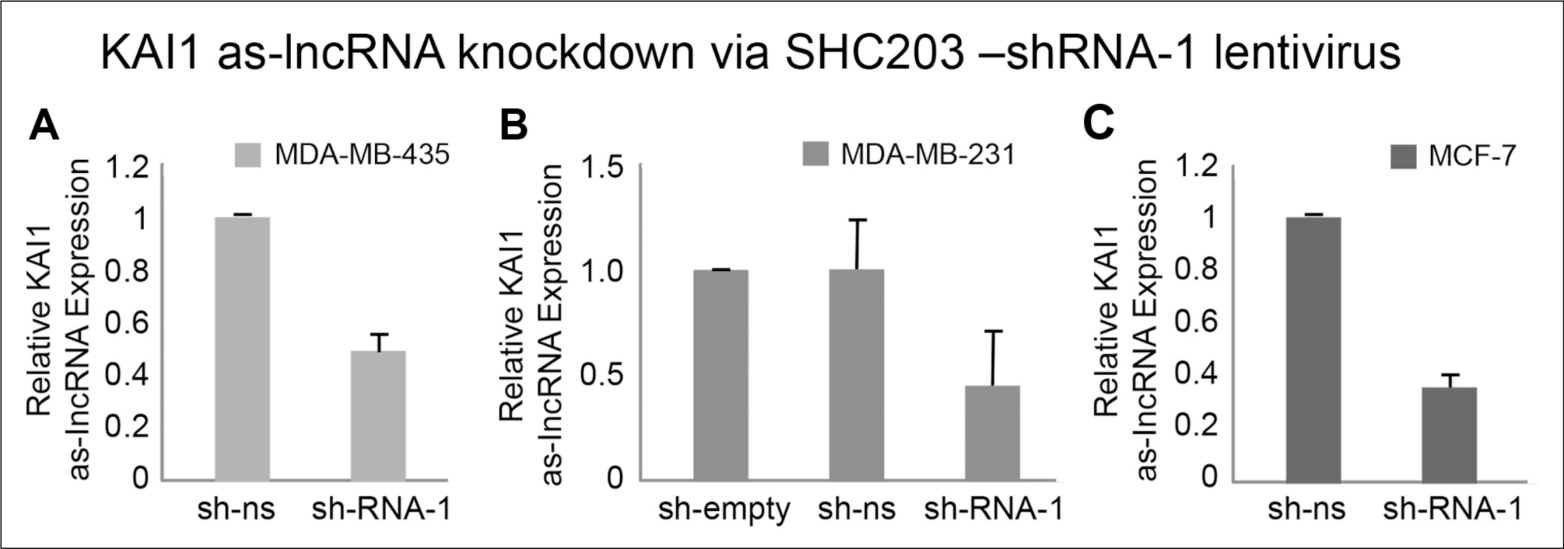
Quantitation of KAI1 as-lncRNA following its shRNA mediated knockdown in MDA-MB-231, MDA-MB-435 and MCF-7 cells qRT-PCR after MDA-MB-435 **(A)**, MDA-MB-231 **(B)** and MCF-7 cells **(C)** infection with SHC-shRNA vector empty, SHC-shRNA- non-silencing (ns) or shRNA-1 against KAI1 as-lncRNA (SHC-shRNA-1). All samples were reverse transcribed with RevertAid Premium RTase (or without RTase as control). Results are average of five independent experiments in triplicates. HPRT-1 mRNA served as endogenous control. The mean of sh-ns was set to 1 in panels (A & C), with the mean value of sh-empty set to 1 in panel (B). *p* (“SHC-shRNA-1”) <0.001 in all three cell lines.

As shown in Figures 4A-4C, compared to the non-silencing vector in MDA-MB-435, MDA-MB-231 and MCF-7 cells, there were 53%, 55% and 63% knockdowns of the KAI1 as-lncRNA, respectively. Introduction of the SHC empty vector control have shown that the value obtained by SHC non-silencing shRNA vector was equivalent to the former, not displaying any drop in KAI1 as-lncRNA (Figure 4B).

**Figure 5:**
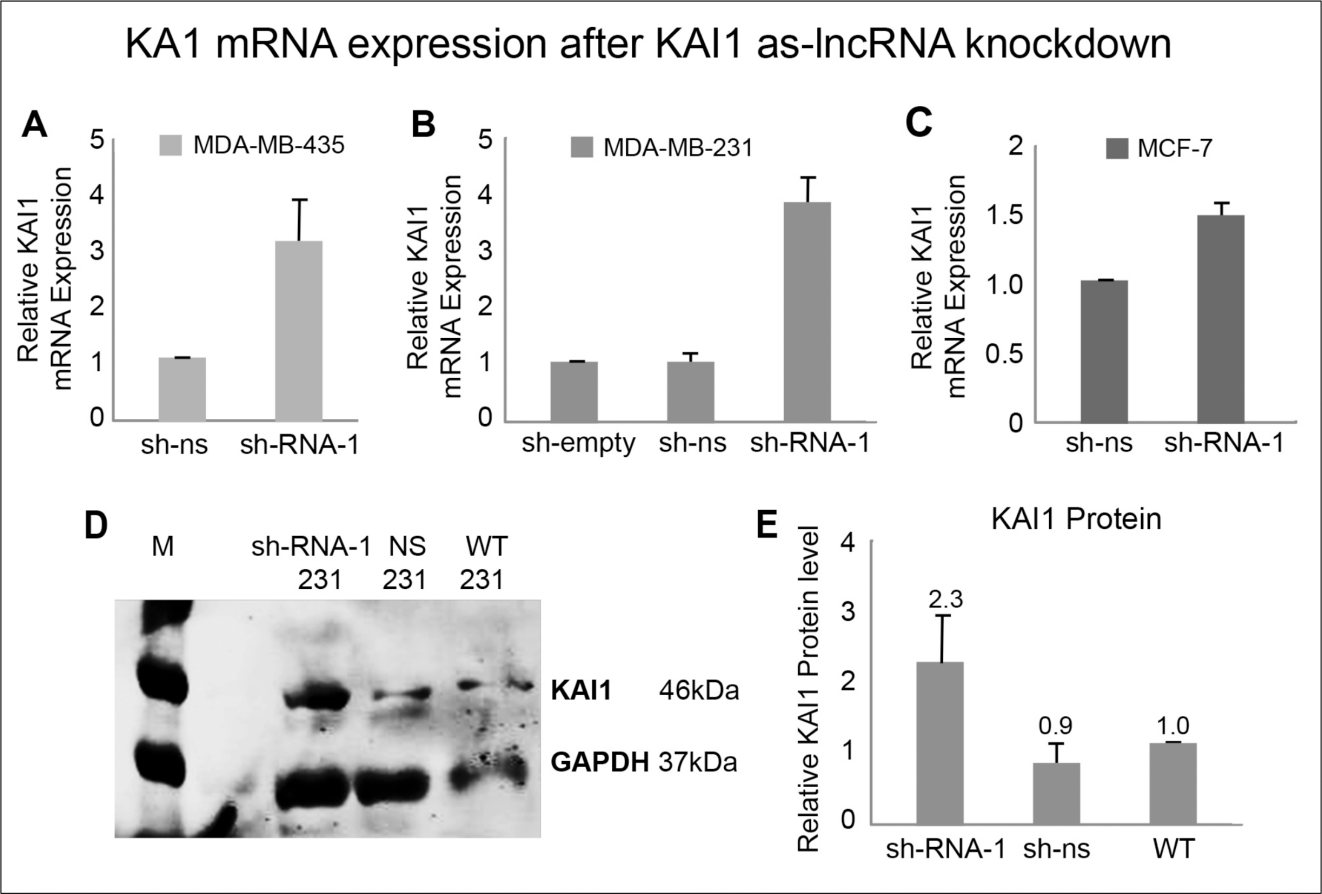
KAI1 mRNA and protein expression following KAI1 as-lncRNA knockdown MDA-MB-435 **(A)**, MDA-MB-231 **(B)** and MCF-7 cells **(C)** were infected with SHC-shRNA-1 and compared via qRT-PCR/ Western blot analysis to controls for KAI1 mRNA, as well as for KAI1 protein, respectively. Non-silencing shRNA or SHC empty vector served as controls. Relative KAI1 mRNA expression quantified by Real-Time PCR with HPRT-1 as endogenous control. The mean of sh-ns was set to 1 in panels A & C, with the mean value of sh-empty set to 1 in panel B. Knockdown in panels a-c *p*< 0.01. As for KAI1 overall protein level, the cells total proteins were lysed in 2% CHAPS. 80 μg protein were loaded on 10% SDS-PAGE gel, immunostained with C-16 anti KAI1 and FL-335 anti GAPDH (both Santa Cruz) and detected using the Odyssey Infrared Imager **(D)**. Quantitative analysis was performed with ImageJ Software representing an average of three independent experiments **(E)**. Setting the WT value to 1, knockdown in panel E *p*<0.05.

After this partial knockdown of KAI1 as-lncRNA in these three cell lines was established, the knockdown effect, if any, on the expression level of KAI1 mRNA was examined (Figures 5A-5C). While using the value obtained with NS-shRNA as the reference point (1.0), knockdown of KAI1 as-lncRNA via SHC-shRNA-1 resulted in ∼4 fold increase of KAI1 mRNA in MDA-MB-231 and MDA-MB-435 cells, with only 1.5 fold in MCF-7 cells.

The shRNA-empty control did not have any influence on either the KAI1 as-lncRNA or its mRNA expression level.

### The influence of KAI1 as-lncRNA knockdown on the KAI1 protein

Next, we examined whether the increased KAI1 mRNA expression in virus infected cells, following knockdown of the KAI1 as-lncRNA, also results in an elevated protein level. As demonstrated in Figure 5D & 5E, indeed knockdown of the KAI1 as-lncRNA with SHC-shRNA-1 led on the average to 2.3 fold higher KAI1 protein level, compared to the control wild type MDA-MB-231 cells, or to those infected with non-silencing shRNA expressing SHC virus.

Yet, in order to be sure that knockdown of the KAI1 as-lncRNA was not due to an artifact of the lentiviral virus SHC203 itself, a similar test using another lentiviral vector was performed. To this end, the pTRIPZ doxycycline-induced lentiviral vector (Open Biosystems Inc.) was used for cloning of shRNA-1 and the shRNA-scrambled “non-silencing”. It should be pointed out that whereas TRIPZ had a 19-nucleotide hairpin loop, the SHC203 vector harbored only 6 bases hairpin loop; yet, the processed active siRNA was obviously the same. Moreover two other shRNAs designed against KAI1 as-lncRNA (Broad-1 & Broad-2) were similarly cloned to the TRIPZ vector (see M & M).

**Figure 6:**
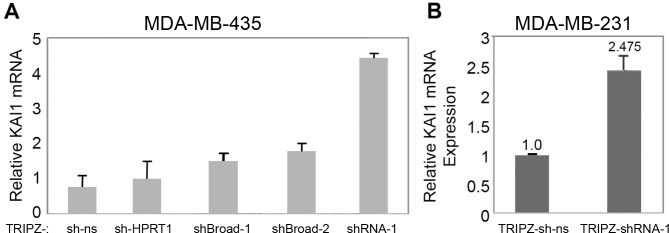
KAI1 mRNA level following KAI1 as-lncRNA knockdown via the TRIPZ lentiviral based vector MDA-MB-435 **(A)** and MDA-MB-231 **(B)** cells were infected with TRIPZ-shRNA-1 (A & B) or with TRIPZ-Broad-1- or TRIPZ-Broad-2 shRNAs expressing lentiviruses (A), to disrupt the KAI1 as-lncRNA (see their structure/construction in M & M). Doxycycline 2.5μg/μl and/or puromycin 0.1-1 μg/ml were added 24 hours post infection. Relative KAI1 mRNA Expression was quantified by Real-Time PCR with HPRT-1 as endogenous control. In panel B knockdown: *p*< 0.01.

Infection with that inducible vector led with shRNA-1 to ∼4.4 fold increase (Figure 6A), and ∼2.5 fold increase (Figure 6B) of the KAI1 mRNA, in MDA-MB-435 and MDA-MB-231, respectively. Interestingly, even though the passenger/target sequences of Broad-1 and Broad-2 closely flank that of shRNA-1, their infected shRNAs led to only 1.5-1.75 fold elevation in KAI1 mRNA of MDA-MB-435 (Figure 6A). Consequently, the rest of the project has been performed with shRNA-1 based-TRIPZ or SHC203-shRNA-1 lentiviruses only.

### The effect of ectopic expression of KAI1/CD82 gene on MDA-MB-231 cell proliferation

It is known that a high proliferation rate due to limitless replicative potential is one of the six hallmarks of cancer cells. However, Malik et al., [20] observed no difference in MDA-MB-231 cell growth kinetics when comparing their KAI1 knockdown, KAI1 overexpression and wild type untreated cells. To make sure on this phenotypic important point, the KAI1 open reading frame cDNA was amplified and cloned into the doxycycline-inducible lentiviral expression vector TRIPZ in sense or antisense orientation, as outlined in M & M. The inducible expression of both exogenous KAI1 transcripts, in sense (KAI1^OE^) and antisense (KAI1^OE-AS^) orientation in MDA-MB-231 cells was demonstrated by qRT-PCR. Both exogenous transcripts were expressed in MDA-MB-231 cells, the sense orientated KAI1 ORF RNA about 3.9 fold, the antisense orientated ORF RNA even 6.8 fold more than the endogenous wild type KAI1 mRNA, leading to ∼2.2 fold more KAI1 protein and knockdown of the KAI1 polypeptide, respectively (data not shown). Following, TRIPZ (comprising the sense or antisense ORF encoding fragment) infected MDA-MB-231 cells were seeded in 48 wells and their doubling time was measured every 24 hours, for 4 days. The average doubling time of the four samples was about 20-26 hours (Supplementary Figure 3). In accordance with Malik et al. results [20], no significant difference in the doubling time of KAI1 overexpressing cells (ORF S) compared to ORF AS (the latter under-expressing the KAI1 protein due to over expression of the KAI1 antisense RNA.).

### The phenotype of KAI1 as-lncRNA knockdown cells: wound healing

As indicated above, KAI1 has been reported to play a crucial role in invasiveness and metastasis [8]. This phenotype was further studied, in order to test whether knockdown of KAI1 as-lncRNA and consequently the increased KAI1 mRNA and protein expression, would rescue the metastasis suppressive character of KAI1.

**Figure 7:**
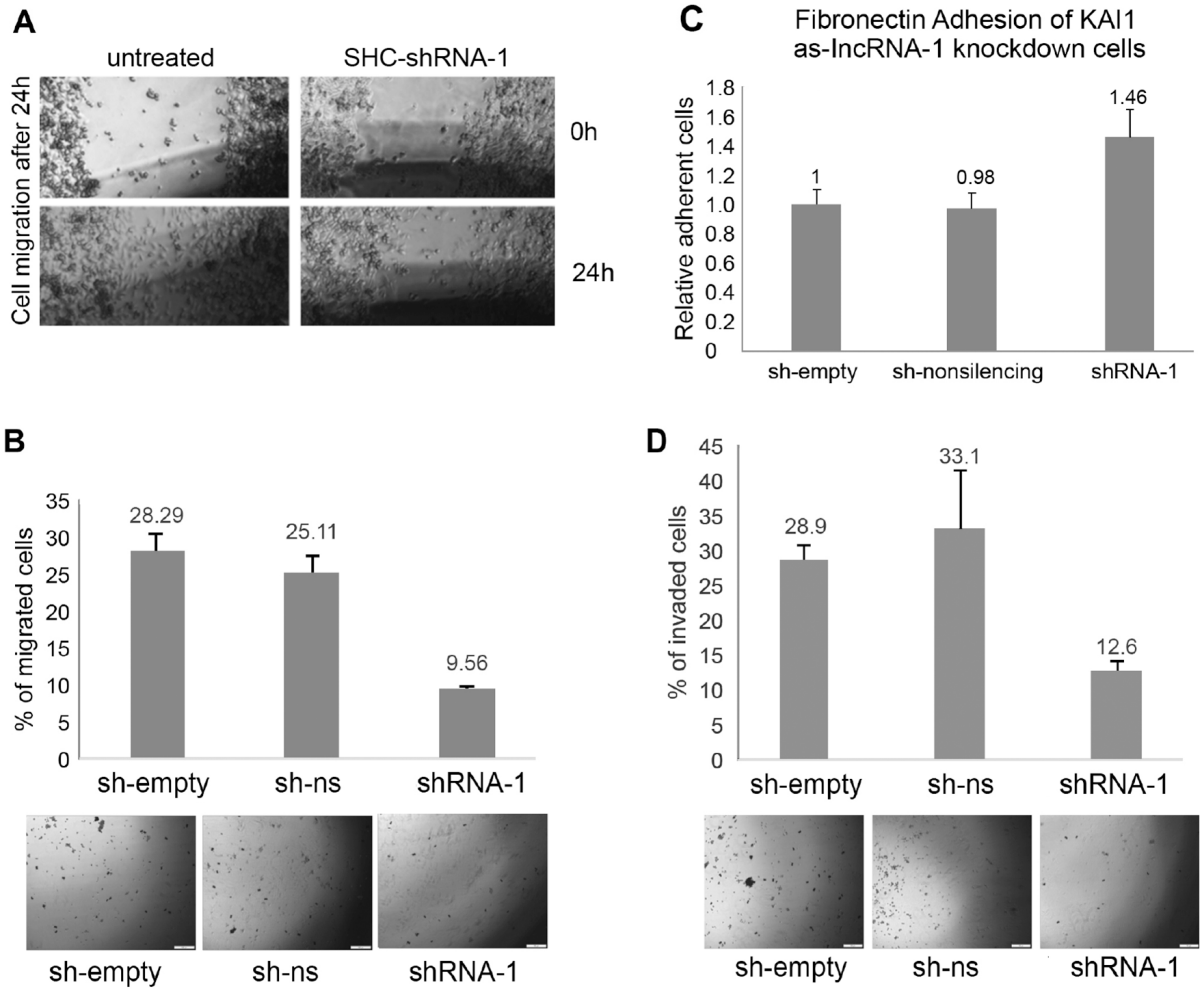
Phenotypic analysis of MDA-MB-231 knocked down for KAI1 as-lncRNA following SHC-shRNA-1 lentiviral infection **(A)** *in vitro* scratch wound healing in untreated MDA-MB-231 cells or MDA-MB-231 cells infected with SHC-shRNA-1 lentivirus. Images captured using light-field microscope after 0 and 24 hours represent four repetitions with duplicates. **(B)** migration through uncoated transwell chamber. Percent of invaded cells calculated by mock migration control. Images were taken by light-field microscope camera as illustrative support. *p* “SHC-shRNA-1” <0.05 (two sample t-test). **(C)** adhesion to fibronectin coated plates, as compared to cells infected by SHC-empty lentiviral vector. Average of three independent experiments using triplicates as samples. *p*-value for “SHC-shRNA-1” =<0.01(two sample t-test). **(D)** Invasion through Matrigel coated transwell chamber. Percent of invaded cells calculated by mock invasion control. Images were taken by light-field microscope camera. *p* “SHC-shRNA-1” <0.05 (two sample t-test).

The mechanism of tumor dissemination by both metastasis and invasion is based on the cell motility machinery. It has been mentioned above that elevating KAI1 expression leads to retardation of cell mobility of cancer cells *in vitro*. Therefore, a scratch wound healing assay was performed on untreated MDA-MB-231 cells vs. same cells infected by SHC203-shRNA-1 based viruses targeting the KAI1 as-lncRNA, at zero time and 24 hours thereafter. As demonstrated in Figure 7A, after 24 hours the wounds of untreated cells healed, while the wound healing of shRNA-1 expressing cells was noticeably retarded. The cells migration-retardation is not due to the effect of SHC203 lentiviral infection by itself because sh-empty and shRNA-non-silencing expressing viruses did not elicit such wound healing inhibition (data not shown). Thus the KAI1 as-lncRNA induced retardation of cell migration is most likely caused by the resulting KAI1 enhancement.

### The phenotype of KAI1 as-lncRNA knockdown cells: cell-migration, -adhesion and -invasion

Similar results were obtained, when performing a trans-well (Boyden chamber) migration assay. In line with the scratch wound healing assay, less SHC-shRNA-1 infected cells migrated (∼9.5%) compared to the control cells infected with either an empty vector (sh-empty) or a scrambled shRNA (sh-non-silencing) of which 28% and 25% of the cells migrated through the trans-well, respectively (Figure 7B). Both the wound healing assay and the migration assay through a trans-well showed that increased KAI1 mRNA and thereby increased protein expression, due to KAI1 as-lncRNA knockdown, resulted in migration-retardation of MDA-MB-231 cells *in vitro*.

It is known that carcinoma cells that become metastatic have to disconnect from the original cell mass. Thus, loss of cell-cell adhesion can be the initial step of cell dissemination. It has been reported that KAI1 mediates cell-cell adhesion by altering the stability and density of alpha-integrins [8]. In order to test the adhesion of MDA-MB-231 cells after KAI1 as-lncRNA knockdown, a fibronectin adhesion assay was performed.

The results, which are illustrated in Figure 7C, indicate around 46% stronger adhesion to fibronectin coated culture dishes by cells infected with shRNA targeting the KAI1 as-lncRNA (SHC-shRNA-1) as compared to the control infections (the sh-empty control is set to 100%).

It has been reported that KAI1 acts as a metastatic suppressor that decreases invasion. Breast cancer cells that disconnect from the primary tumor cell mass have to be able to degrade extracellular matrix components in order to invade normal surrounding tissue. As shown in Figure 7D, knockdown of the KAI1 as-lncRNA led to > 50% less invading cells compared to the empty vector and non-silencing controls.

The observed effect of KAI1 as-lncRNA knockdown on the invasiveness of MDA-MB-231 cells was confirmed by subjecting KAI^OE^ - or KAI1^OE-AS^ - expressing cells to the same invasion assay (Supplementary Figure 4). Induction of the KAI1 mRNA expressing TRIPZ vector in MDA-MB-231 led to a ∼62% reduced invasion rate in comparison to the non-induced cells. In contrast, induced KAI1 mRNA expression in antisense direction created a highly invasive phenotype (as outlined above for the migration assay), which is likely to reflect consequences of KAI1 mRNA degradation/inhibition.

Guttman and Rinn reported that lncRNAs can act as scaffolds to bridge protein or protein complexes with chromatin regions [21]. Since the KAI1 as-lncRNA overlaps the enhancer region of the KAI1 promoter, perhaps this transcript is involved in mediation/inhibition of the transcription factor-DNA interaction, leading to decreased expression of the sense KAI1 mRNA transcript. This might indicate a regulatory role of the KAI1 as-lncRNA.

Moreover, these results suggest an oncogenic like activity of the KAI1 as-lncRNA as we have demonstrated how shRNA mediated knockdown of the KAI1 as-lncRNA elevates the expression of the silenced KAI1 gene, which in turn rescues the metastatic suppressive attributes of KAI1. The fact that shRNA mediated degradation of as-KAI1 leads to enhancement of KAI1 expression, strongly suggest a cis-acting RNA mechanism [22]. Based on these results we have named this newly identified lncRNA- SKAI1BC, as in “Suppressor of KAI1 in Breast Carcinoma”. Expression of SKAI1BC lncRNA might be used as a potential biomarker for tumor prognosis (aggressiveness) in the future. Noteworthy, recently the FDA has approved the usage of the PCA3 lncRNA as a biomarker for prostate cancer, while being sampled from the urine [23, 3].

The re-expression in TNBC derived cell lines of the epigenetically silenced metastasis suppressor gene KAI1 brings up the formal possibility of the metastasis regulatory lncRNA SKAI1BC as a therapeutic target in TNBC. Noteworthy, the fact that partial inactivation of SKAI1BC alone has such dramatic consequences on breast cancer metastatic features, (as yet in *in vitro* cultured human cancer-derived cells), is therapeutically promising.

Due to the prevalence of KAI1/CD82 gene down regulation in at least twelve other human cancers, perhaps SKAI1BC lncRNA targeting is relevant for therapy of additional human malignancies.

Supplementary Data are available at Oncotarget Online.

## MATERIALS AND METHODS

### Cell culture

The cell lines MDA-MB-231, MCF-7 and HEK293T were received from Prof. L. Vardimon, R. Pinkas, and Y. Shiloh, respectively (Tel Aviv University). Hs578T was obtained directly from ATCC, while MDA-MB-435 was received from Prof. J. Price (MD Anderson, Houston), and SUM149PT from Prof. J. Hoheisel (DKFZ, Heidelberg). Cell line authentication was performed by determination of their short tandem repeat (STR) profile (Promega PowerPlex 16 HS kit). The cells were also routinely checked for *mycoplasma* with EZ-PCR Test Kit (Biological Industries, Israel). The cell lines were cultured at 37°C in 5% CO_2_ in full Dulbecco's modified Eagle medium (DMEM) supplemented with 10% fetal bovine serum (FBS) (Sigma-Aldrich), 4mM L-glutamine, sodium bicarbonate (Biological Industries), 10 units/ml of penicillin and 50μg/ml streptomycin. HEK293T human embryonic kidney cells were kept under G418 antibiotic selection pressure to maintain the SV40 large T-Antigen for increased production of replicons harboring SV40 origin. Culture medium for lentiviral transduced cells was supplemented with puromycin as a selective drug.

### Plasmids pGEM^®^-T easy cloning

PCR products amplified with Taq-polymerase, which leaves a thymine, adenine (TA)-overhang, were ligated with T4 DNA Ligase into pre-digested 50 ng pGEM^®^-T Easy vector (Promega), according to manufacturer's instructions. Afterwards the ligation mixture was either transformed into CaCl_2_-competent *E.coli* or electroporated into electro competent *E.coli* cells.

### Lentiviral plasmids

The doxycycline Tet-on inducible lentivirus TRIPZ (Open Biosystems) served as expression vector as follows: Empty TRIPZ vector; TRIPZ-Non-Silencing (having the nucleic acid sequence: ATCTCGCTTGGGCGAGAGTAAG representing the 22 bases no target-like sequence); and an shRNA termed herein shRNA-1 having the 21 bases target-like/passenger sequence ACAACTCATGGGTACTCTCGT (all present in the 792 bp long KAI1 as-lncRNA) followed by 19 nucleotide hairpin loop TAGTGAAGCCACAGATGTA, and the 21 bp complementary inverted repeat guide sequence ACGAGAGTACCCATGAGTTGT. Likewise, we built into the TRIPZ vector two additional shRNA encoding oligonucleotides based upon an algorithm developed at Broad Institute and called appropriately Broad-1 and Broad-2 whose target like sequence being Broad-1: 21 bp passenger/target-like sequence CTCTATGCCAAGCGCTGAATA followed by the 19 bp loop and the 21 bp complementary inverted repeat guide sequence TATTCAGCGCTTGGCATAGAG. Likewise Broad-2 has 21 bp passenger/target-like sequence TTTACTAATGCGCAGTTTAAG followed by the 19 bp loop and the 21 bp complementary inverted repeat guide sequence CTTAAACTGCGCATTAGTAAA. The constitutive Lentiviral Vector SHC203 (Sigma)

derived from the pLKO.5 vector with a puromycin resistance gene and a turboGFP reporter, served as expression vector for the following shRNAs: Empty; shRNA Non-Silencing; and shRNA-1 expressed from a U6 promoter, and having a loop of 6 nucleotides (CTCGAG) rather than the 19 present in TRIPZ. The TRIPZ expression vector also served to express the 804 bp Open Reading Frame of the KAI1 protein which was prepared as follows: MDA-MB-231 total RNA was reverse transcribed with oligo dT primer and amplified by Q5 TAQ Polymerase (NEB) with KAI1 cDNA specific primers: KAI1 ORF Forw. ATGGGCTCAGCCTGTATCAAAG and KAI1 ORF Rev. TCAGTACTTGGGGACCTTGCTG. The ORF containing fragment was cloned into a TRIPZ vector digested with EcoRI and AgeI (to cut off the reporter marker turboRFP and the shRNA-non-silencing, so as to enable the virus packaging) and ligated with an adapter containing the restriction site SnaBI.

### SHC203 and TRIPZ lentiviral shRNA production and infection

Both vectors are replication-defective lentiviruses of the second generation and were produced in HEK293T cells. Each vector (shRNA containing vector) was co-transfected with two second generation packaging plasmids, psPAX2 and pMD2.G, (kindly given to us by Trono’s laboratory) that encode for the viral helper proteins. Experimental details for the viruses’ generation and further infection of breast cancer cell lines, as outlined [24, 25].

### RNA pull down with magnetic dynabeads^®^

Total RNA (2-60 μg) with 1-5 pmole Biotinylated-Oligo(dT)_25_ were heated for 1 minute at 70°C to open up secondary structures and snap chilled on ice. NaCl (1M) and EDTA (10 mM) were added to a final concentration of 200 mM and 0.2 mM, respectively. Annealing was allowed at room temperature under steady rotation for 30 minutes. Meanwhile, 2-3x molar excess of super magnetic beads (Thermo Fisher Scientific M-280), which are covalently coupled to a monolayer of recombinant streptavidin, were prepared for RNA manipulations by washing with 0.lM NaOH and 0.05M NaCl. The RNA-biotinylated-oligomer complex was added to the magnetic beads and rotated at room temperature for 30-60 min. The very high binding affinity of the biotin-streptavidin interaction (K_d_= 10^−15^) permits the specific isolation of the annealed polyA plus RNA by placing the tube on a magnet, while saving the polyA minus RNA containing supernatant. The magnetic beads bound polyA plus RNA was eluted in 1mM Tris (pH 8) and 0.1mM EDTA (pH 8) and used for downstream applications.

### 5'- RACE

To determine the transcription start of the polyA minus lncRNA, 60 μg RNA was annealed to a 3'-biotinylated −60 mer and bound to streptavidin coupled magnetic beads. Following, the RNA was reverse transcribed with a transcript specific primer, RNase A treated and polyA tailed by Terminal transferase (Thermo Fisher Scientific) according to the suppliers' instructions. The polyA tailed cDNA was ethanol precipitated and cleaned up through a NucleoSpin^®^ Column (Macherey-Nagel). Second strand synthesis with RevertAid Premium (Thermo Scientific) was performed using an adapter with a polyT tail to prime the generated polyA tail of the cDNA. PCR was performed with the adapter (without the polyT tail) and transcript specific primer that served as RT-primer. “Nested”-PCR was performed with the adapter and a transcript specific oligomer priming closer to the 5'-end of the cDNA transcript. PCR products were cloned into pGEM^®^-T Easy Vector and sent to sequencing.

### Transfections and dual-luciferase reporter assay

Transient Transfection of HEK293T cells was performed using jetPEI transfection reagent (Polyplus Transfection), while that of MDA-MB-231 and MCF-7 cells with Lipofectamin 2000 (Thermo Fisher Scientific) according to the manufacturer instructions. If induction or selection of the transfected DNA for stable transfectants was desired, 2.5μg/μl doxycycline and/or 0.1-1 μg/ml puromycin were added 24 hours post transfection, respectively. As for the luciferase reporter assay (see ref. 26), the pGL3-basic Vector from Promega (a kind gift from L. Vardimon lab) was digested with SmaI and ligated with the PCR amplified (Q5 polymerase) 1198bp KAI1 promoter-enhancer region in sense and antisense orientations. The pGL3-basic, pGL3-basic-promoter sense and pGL3-basic- promoter antisense were co-transfected with TK-Renilla in a 1:20 ratio, respectively. Forty eight hours post transfection, cells were harvested and lysed in 1x passive lysis buffer. Firefly and *Renilla* luciferase activities were measured using an LKB Wallac 1250 Luminometer. The firefly luciferase luminescence measured was proportional to the promoter activity driving it. The *Renilla* luciferase luminescence was proportional to the efficiency of the transfection serving as an internal control. Normalized luciferase luminescence was calculated as followed: [(firefly luciferase activity/Renilla luciferase activity)×100].

### RNA extraction from cell culture

Cells were detached with Trypsin-EDTA from their culture dish, centrifuged for 1 min at 2000 rpm in a table biofuge (Heraeus). To extract total RNA, EZ-RNA Kit (Biological Industries, Israel) was used according to the manufacturer instructions. After RNase free RQ1 DNase treatment (Promega) total RNA was subjected to sodium periodate treatment as outlined by Tzadok et al., 2013 [in ref. 10].

Nuclear RNA was isolated by perforating the cells with 0.1% digitonin in Hank’s Balanced Salt Solution (HBSS) and 1mM PMSF. Following, cell ghosts were collected by a low speed spin. The pellet was further subjected to RNA extraction, with the EZ-RNA kit (Biological Industries).

### Reverse transcription

Reverse Transcription was carried out using 200 U RevertAid^TM^ Premium (Thermo Fisher) which is an RNase H minus M-MLM derived enzyme, at 50-60°C (when using gene specific primer), or at 42°C when using random hexamer or Oligo dT_(15)_ primer. RT with 30 U AMV derived enzyme (Thermo Fisher) were carried out at 37°C. Usually, 1 μg of RNA was subjected to reverse transcription reaction in 20 μl. The RT reaction was then diluted to 100 μl with DEPC treated water. RT reactions (5 μl) were subjected to PCR amplification using Maxima Hotstart Taq- polymerase (Thermo Fisher). PCR conditions were as follows: 95°C for 4 min for initial denaturation and activation of the hotstart enzyme, 25-40 cycles of 95°C for 30 seconds, Tm −5°C for 30 seconds for annealing and 72°C for 30 sec/kb for elongation. A final extension step at 72°C for 4 min was applied for most amplifications. Actinomycin D was added to the reverse transcription reaction at a concentration of 50 ng/μl. Following RT, for the PCR amplification step, Actinomycin D had to be diluted to 1.25 ng/μl in order not to inhibit the Taq Polymerase activity.

### Quantitative real-time PCR

All reactions were performed in 20 μl mixtures consisting of 2x Quanta perfect SYBR Green fast mix (Quanta Bioscience), 10 pmole of respective primers and 25-150 ng of cDNA. HPRT-1 served as internal reference for stable transcripts. Amplification conditions were as follows (if not stated differently): Initial incubation at 50°C for 2 min. and 95°C for 3min., followed by 40 cycles of 95°C for 10 seconds, 60-61°C for 30 seconds and a final dissociation stage (2 cycles of 95°C 15 sec. and 60°C 1 min.). Calibration curves were performed for every product and primer efficiency was determined, tolerating a range of 95%-105%. Ct values were monitored and analyzed by the 7300 fast Sequence Detection System Software (Applied Biosystems). All reactions were performed in triplicates.

### Samples preparation for western blot analysis and SDS-PAGE followed by electro-blotting

This part was carried out according to Gallagher’s laboratory [27, 28].

### Imaging

The Nitrocellulose membrane was blocked for 60 minutes with Odyssey^®^ Blocking Buffer PBS (LI-COR) at room temperature under constant shaking. After blocking, an overnight incubation with the primary antibody at 4°C followed. The primary polyclonal antibody C-16 (Santa Cruz) raised in rabbits against human CD82 (KAI1) was diluted 1:200 in Odyssey^®^ Blocking Buffer. On the next day, the membrane was washed three times with PBS-Tween before incubation with the DyLight 800 (KPL) secondary antibody (goat anti rabbit) for 60 minutes at room temperature, diluted 1:10,000 in Odyssey^®^ Blocking Buffer. After three washes with PBS-tween, the membrane was exposed to infrared detection with the Odyssey^®^ Infrared Imager (LI-COR). Similarly, immuno-staining with endogenous control antibody FL-335 (rabbit anti human GAPDH; 1: 1,000, Santa Cruz) was for two hours at room temperature followed by secondary antibody DyLight680 (KPL), washing and exposure as outlined above.

### Cell growth assay

Cells were seeded at various concentrations in a 96-well plate in four respective triplicates. On day 0 the cells of the first triplicate series were exposed to Resazurin Cell Viability Assay. Cell Viability was determined 24, 48 and 72 hours later with the respective next series. Doubling time was calculated by comparing cell viability at day 1 to day 0, day 2 to day 1, etc.

### Wound healing assay

Monolayer cells were grown to 100% confluency and deprived from serum 24 hours prior to the assay. With a plastic pipette tip a scratch was inflicted to the monolayer. Cells were carefully washed with TS and serum free RPMI 1640 medium was added. Random fields of the scratch wound were marked and photographed with a light microscope camera at time 0 and after 24 hours incubation at 37°C with 5% CO_2_.

### Migration assay

To study cell migration, ThinCert™ (Greiner Bio-One) cell culture inserts were placed in a multi well cell culture plate. The insert contains a polyethylene terephthalate (PET) membrane at the bottom with a pore size of 8 μm that separates the upper from the lower compartment. Then 100,000 serum-starved cells were seeded at the top of the insert in 200 μl serum free media, while the lower compartment contained 10% FBS DMEM media as a chemoattractant that may induce active migration of the seeded cells through the PET membrane. Adherent cells that migrate through the pores remain attached to the under-side of the PET-membrane. After 24 hours, the medium in the inserts and lower compartment was removed, the inserts washed with TS and incubated with Trypsin-EDTA for 10 minutes at 37°C to detach the migrated cells from the under-side of the insert. The migrated cells were seeded in 48-well plate. To estimate the percentage of migrated cells, a Resazurin Cell Viability Assay was performed and compared to the mock control cells.

### Fibronectin adhesion assay

Cells grown in 10% FBS containing DMEM were deprived from serum 24 hours prior to the adhesion assay. Forty eight well plates were coated with 10 μg/cm^2^ fibronectin diluted in HANK's Balanced Salt Solution and incubated overnight at 4°C. On the next day fibronectin coated 48-well plates were washed three times with PBS and blocked with 10mg/ml heated BSA in PBS for 30 min. Cells were then collected, washed twice in PBS and resuspended in RPMI 1640 medium. Then 50,000, 100,000 or 200,000 cells were seeded in fibronectin coated and uncoated wells, respectively, and incubated for 30 minutes at 37°C. After 30 minutes, unattached cells were washed away. On the next day, cell viability was determined by Resazurin Cell Viability Assay and percent of adherent cells was calculated relative to control wells.

### Invasion assay

The ability of cells to invade through extracellular matrix was studied in an invasion assay (29, 30). Therefore, the same method of the ThinCert^™^ Migration assay was applied with the only difference being that the ThinCert^™^ inserts had to be coated with basement membrane matrix (Matrigel, Sigma). For coating, Matrigel had to be thawed on ice and diluted in serum free media to 1μg/μl and pipetted to the center of the PET-membrane. The coated membrane was incubated at 37°C for 4 hours and air dried under sterile conditions.

### Statistical analysis

Statistical significance was evaluated using the one sample t-test against the null hypothesis that the expectation is 1. In Figure 7 panels B-D an unpaired two sample t-test was performed.

## SUPPLEMENTARY MATERIALS FIGURES AND TABLES


