# Supplementary material for: Identification of a novel metastasis inducing lncRNA which suppresses the KAI1/CD82 metastasis suppressor gene and is upregulated in triple-negative breast cancer
Source: Oncotarget. 2017 Jun 28;8(40):67538–52. doi: 10.18632/oncotarget.18733 (PMC5620192; doi:10.18632/oncotarget.18733)
Supplement: Supplementary file 1 [file oncotarget-08-67538-s001.pdf]

Identification of a novel metastasis inducing lncRNA which suppresses the KAI1/CD82 metastasis suppressor gene and is upregulated in triple-negative breast cancer

SUPPLEMENTARY MATERIALS

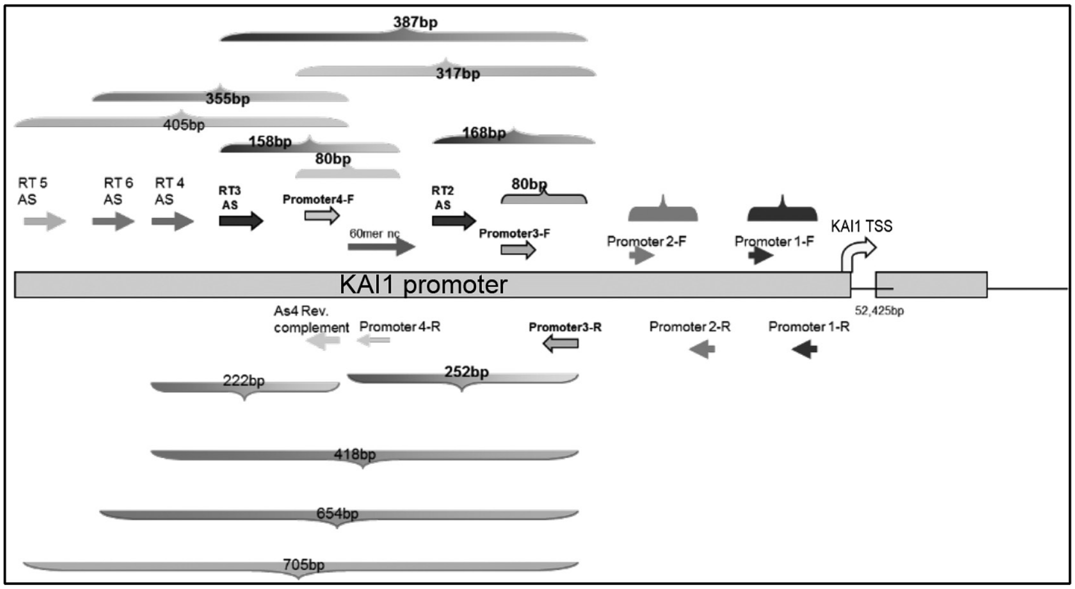

Supplementary Figure 1: Scheme of primers location on the KAI1 as-lncRNA transcript. Arrows indicate position of designed primers in either sense or antisense orientation, with sizes of the resulting amplicons (above and below).

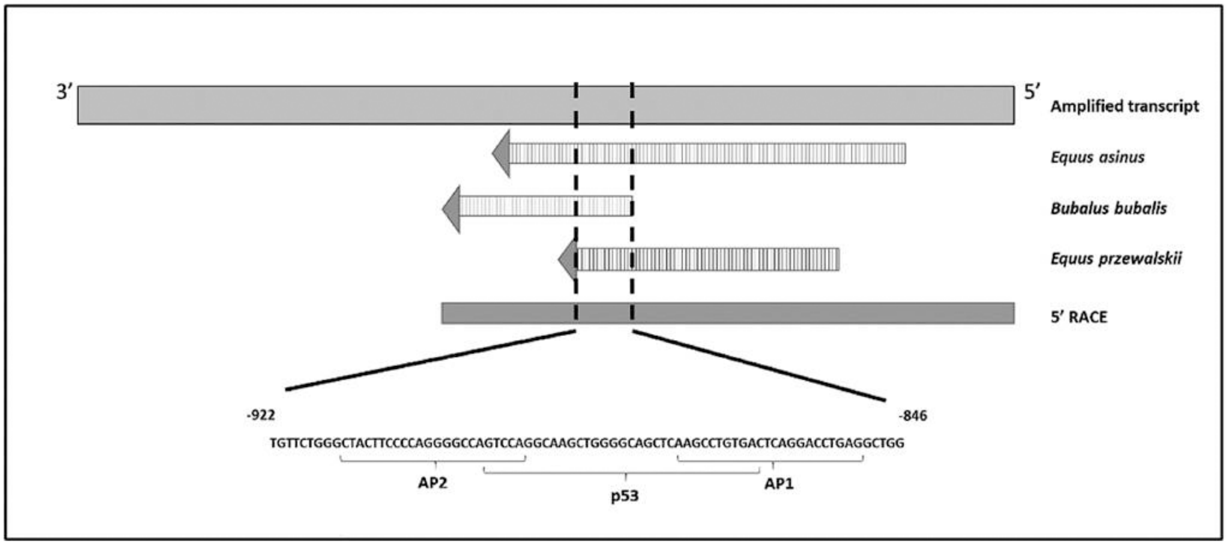

Supplementary Figure 2: Mammalian transcripts homologue to KAI1 as-lncRNA. Schematic illustration of the characterized KAI1 as-lncRNA transcript (top) and relative position of homologue transcripts found in Equus asinus, Equus przewalskii and Bubalus bubalis (middle three lines). 5' end of KAI1 as-lncRNA product (bottom line), with 76 nucleotide DNA sequence mutual to all transcripts known as the KAI1 enhancer with its transcription proteins binding sites.

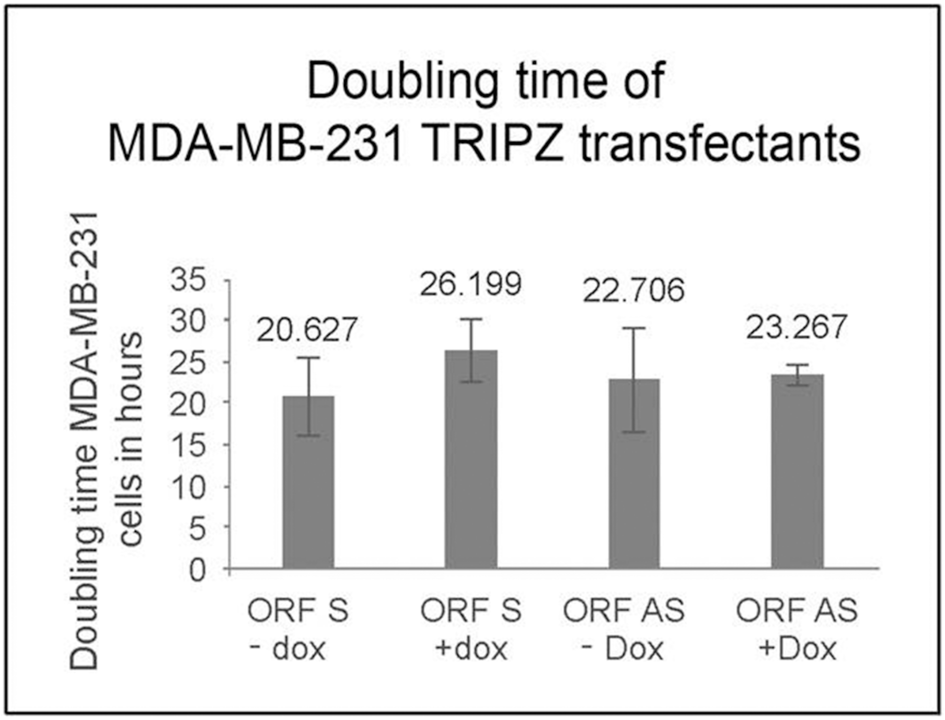

Supplementary Figure 3: Doubling time of MDA-MB-231 cells after KAI1 overexpression. MDA-MB-231 cells infected with the lentivirus TRIPZ-KAI1 ORF in sense or antisense orientation, under doxycycline induced (+dox) or noninduced (-dox) conditions. Doubling time tracked by resazurin cell viability assay over four days.

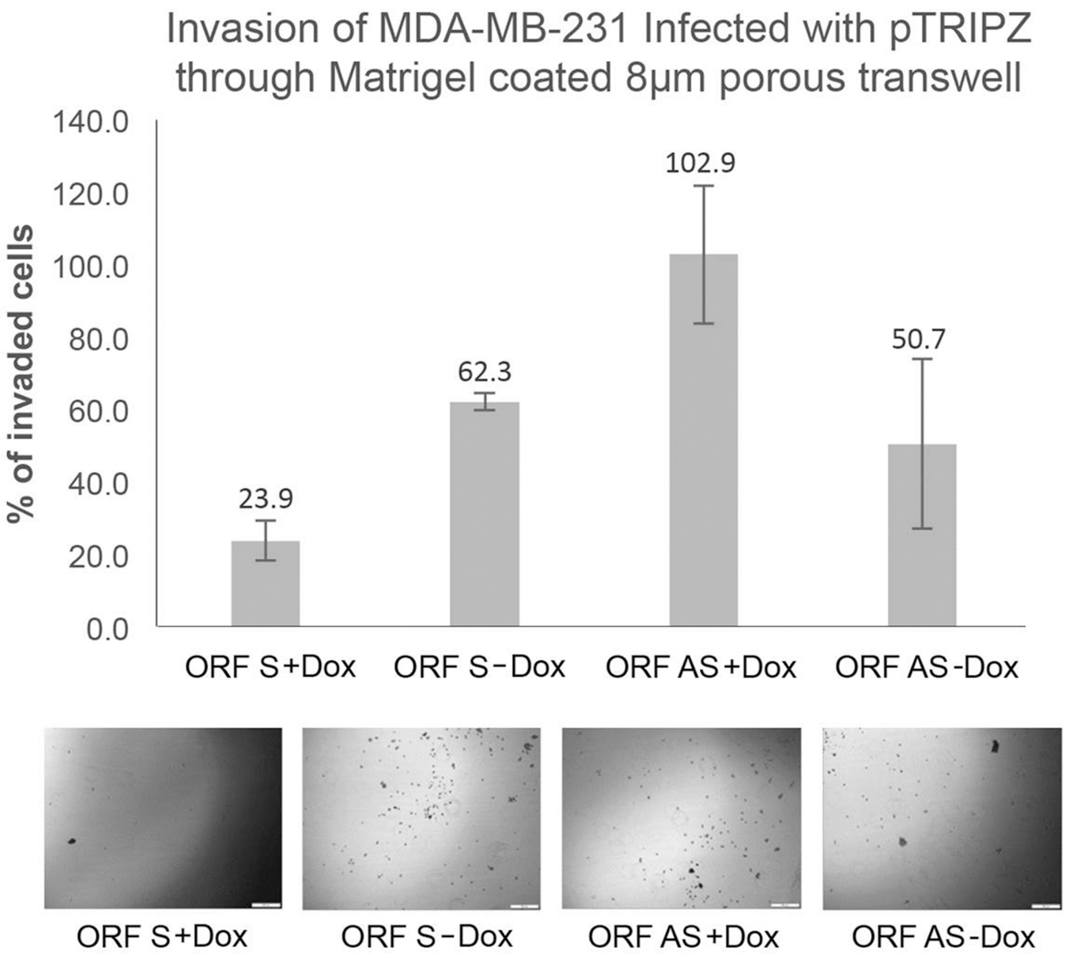

Supplementary Figure 4: Invasion Assay with KAI1 Overexpressing MDA-MB-231 cells. Induced or non-induced MDA-MB-231 cells infected with respective TRIPZ- KAI1 OE expressing viruses and seeded in Matrigel coated trans-well chamber. Cells invading from upper chamber to chemoattractant (10% serum) in lower chamber were detected using resazurin cell viability assay. Percent of invaded cells calculated by mock invasion control. Images were taken by light-field microscope camera.
